# Favorable and poor prognosis B‐cell precursor acute lymphoblastic leukemia subtypes reveal distinct leukemic cell properties when interacting with mesenchymal stem cells, differentially modifying their cell stemness and leukemia chemoresistance

**DOI:** 10.1002/ccs3.70009

**Published:** 2025-06-12

**Authors:** Santiago Ángel Cortés, Paula‐Manuela Rojas Zambrano, Jean‐Paul Vernot

**Affiliations:** ^1^ Fisiología Celular y Molecular Facultad de Medicina Universidad Nacional de Colombia Bogotá Colombia; ^2^ Instituto de Investigaciones Biomédicas Facultad de Medicina Universidad Nacional de Colombia Bogotá Colombia

**Keywords:** adipocyte differentiation, drug sensitivity, leukemic aggressiveness, leukemic niche, MSC reprogramming, multipotent differentiation, senescence

## Abstract

The development of B‐ALL alters the bone marrow microenvironment influencing disease progression and response to therapy. The aggressiveness of particular B‐ALL subtypes could be related to specific mechanisms used to reprogram bone marrow stromal cells. The purpose of this study is to compare the effect of two B‐ALL subtypes, with opposite prognosis, on mesenchymal stem cells (MSC) functions and the consequences on leukemic cell properties. We have established an in vitro leukemic niche (LN) by co‐culturing MSC with REH (favorable prognosis) or SUP‐B15 (poor prognosis) B‐ALL cell lines and examined leukemic‐induced MSC reprogramming and its impact on leukemic cells properties and drug resistance. The aggressive SUP‐B15 cell line showed faster and stronger adherence to MSC and increased migration to CXCL12 and LN secretome, compared to REH cells. SUP‐B15 cell proliferation was reduced but modulated over time. No differences in MSC senescence induction or recovery were observed between both cell lines. Interestingly, the SUP‐B15 LN secretome was enriched in IL‐6, IL‐8, CCL2 and MIF. MSC pre‐incubated with CCL2 showed increased MSC senescence but this did not alter protection against cytotoxic drugs. On the contrary, MSC self‐renewal and adipogenic differentiation were also increased in the aggressive SUP‐B15 cell line, strengthening protection against the cytotoxic drugs vincristine, methotrexate and doxorubicin. This study showed that the aggressiveness of certain leukemia subtypes is also associated with specific changes induced in MSC secretome and stemness that have an impact on specific properties of leukemic cells, improving LN fitness and ability to survive to cytotoxic drugs.

## Graphical abstract

MSC secretome and stemness are altered differentially by B‐ALL cell lines with opposite prognosis. The in vitro model of LN was established by co‐culturing MSC with SUP‐B15 (poor prognosis, left panel) or REH (favorable prognosis, right panel) cells. The most relevant differences found between the two LN, in terms of leukemic properties (adherence, proliferation and migration), MSC functions (differentiation potential and self‐renewal), MSC senescence, LN secretome, and adipogenic‐induced chemoresistance, are shown. Red arrows indicate up‐ or down‐regulation of each assessed feature, compared to controls; = indicates no change.

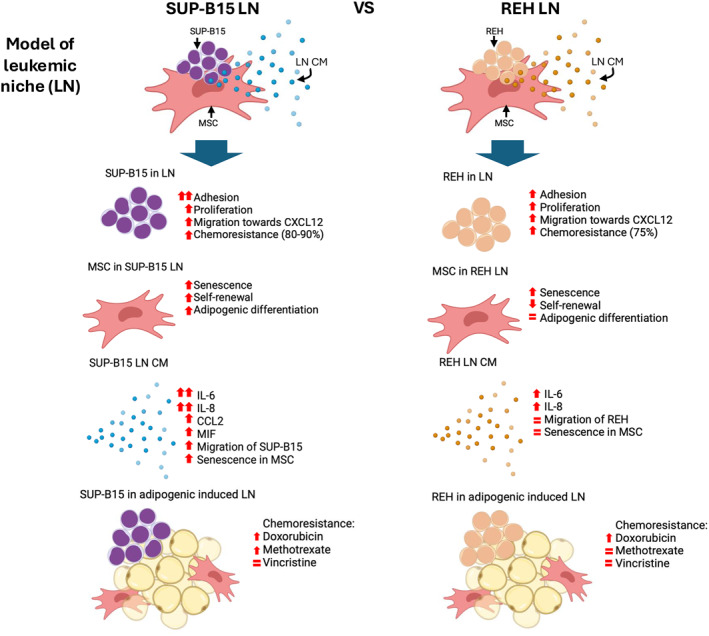

## 1 INTRODUCTION

B‐cell precursor acute lymphoblastic leukemia (B‐ALL) is one of the most prevalent hematological malignancies in the pediatric population. Fortunately, therapies have greatly improved the survival rate in the last 40 years, reaching 80%–90% today.^1^ Nevertheless, even with risk‐stratified and more intensive frontline therapy, 20%–25% of children with ALL still relapsed,^2^, ^3^ the bone marrow (BM) being the main site of regrowth of ALL cells.^4^, ^5^ This preference for BM is due in part to the leukemic cells dependence on both cellular and soluble components present in this modified microenvironment, also called the leukemic niche (LN), which has been early and widely described.^6^, ^7^, ^8^ In particular, mesenchymal stromal/stem cells (MSC) establish a dynamic and bidirectional communication with leukemic cells that modifies not only the functional properties of both interacting cells but also nearby cells including hematopoietic stem and progenitor cells.^9^, ^10^, ^11^, ^12^ Importantly, leukemic cell adhesion to MSC, mainly through integrins and chemokines, and their associated intracellular signaling pathways are responsible for resistance to chemotherapy.^13^, ^14^, ^15^

B‐ALL is a heterogeneous disease with diverse genetic and epigenetic alterations that are known to alter prognosis.^16^ The majority of B‐ALL cases present chromosomal alterations, some of them with favorable (e.g., ETV6‐RUNX1 corresponding to translocation *t* (12; 21) or high hyperdiploidy) or adverse (e.g., BCR‐ABL1 resultant of translocation *t* (9; 22) or hypodiploidy) outcomes.^17^, ^18^, ^19^ In some instances, this aggressive clinical behavior is undoubtedly associated with distinctive biological features of the particular ALL subtype (leukemic cell‐intrinsic properties). This is the case, for instance, of MLL‐rearranged ALL underpinned by stem cell programs, multipotent differentiation capabilities, and a metabolic glycolytic state, inducing higher numbers of leukemia‐initiating cells and lineage switch capabilities.^20^ Nonetheless, increasing evidence suggests that leukemia‐propagating cells occupied and dynamically remodeled preferential LN that may help evading chemotherapy.^21^ It is also important to consider that the remodeling of the mesenchymal LN by leukemic cells was related to heterogeneous clinical prognosis in hematological malignancies.^22^ Accordingly, mesenchymal LN alterations, being an intrinsic self‐reinforcing process of leukemogenesis, may differ depending on the stress induced by a specific leukemia subtype. In particular, differences in MSC functional alterations could influence drastically the survival and maintenance of particular leukemia subtypes or even the selection of particular drug‐resistant subclones. On one hand, this functional heterogeneity may be related to MSC stemness properties (self‐renewal or multipotent differentiation potential) and how these alterations affect LN dynamics (recovery and fitness), and the capabilities of leukemic cells (motility, migration, invasiveness, drug‐resistance, among others), on the other.

We have recently showed in an in vitro LN model (co‐cultures of BM‐MSC with leukemic cell lines or primary culture of leukemic cells obtained from B‐ALL patients), that leukemic growth induced a senescent process in MSC, as evidenced by cell morphology changes (larger and flattened cells with granular cytoplasm), increased senescence‐associated β‐galactosidase (SA‐β‐Gal) activity and expression of cell cycle regulators, probably due to a transient increase in cytoplasmic and mitochondrial reactive oxygen species (ROS).^23^, ^24^ Of note, primary BM‐MSC isolated from B‐ALL patients showed almost the same modifications observed in the LN model, suggesting that senescence induction in MSC is a common process with physiological and clinical relevance.^24^ Intriguingly, the leukemic REH cell line (expressing the fusion protein ETV6‐RUNX1) induced in BM‐MSC an increased expression of p53, while the SUP‐B15 cell line (Philadelphia chromosome positive with the BCR‐ABL1 rearranged gene) produced an increment in p16 expression. The significance of this result is that p53/p21 higher expression has been associated with a reversible senescent process while that of pRb/p16 with an irreversible one.^25^ In fact, we have shown that almost all the changes induced in BM‐MSC by the REH cell line were reversible upon withdrawal of leukemic cells. Yet, some stem cell functions showed greater variability in BM‐MSC isolated from B‐ALL patients than in MSC from the LN model.^24^

In this context, our hypothesis is that the particular aggressiveness of different leukemia subtypes could be related in part to the different mechanisms used to modify the microenvironment, establishing a specific and somehow unique relationship. The peculiarities that may arise are likely to impact (1) the reprogramming of MSC and their ability to cope with or recover from leukemic stress, and (2) the functional properties of leukemic cells. The specific MSC modifications involved, their intensity and persistence, should be relevant to define the LN performance, the leukemic cell properties, and finally the B‐ALL patient outcome. In this work, we have studied whether these two leukemic cell lines (REH and SUP‐B15) with different aggressiveness and prognosis, impact differentially MSC functions with consequences on leukemic cells properties, in particular in their sensitivity to cytotoxic agents commonly used in B‐ALL patients.

## 2 RESULTS

### 2.1 Adherence to MSC is faster and stronger in the aggressive SUP‐B15 cell line

**FIGURE 1:**
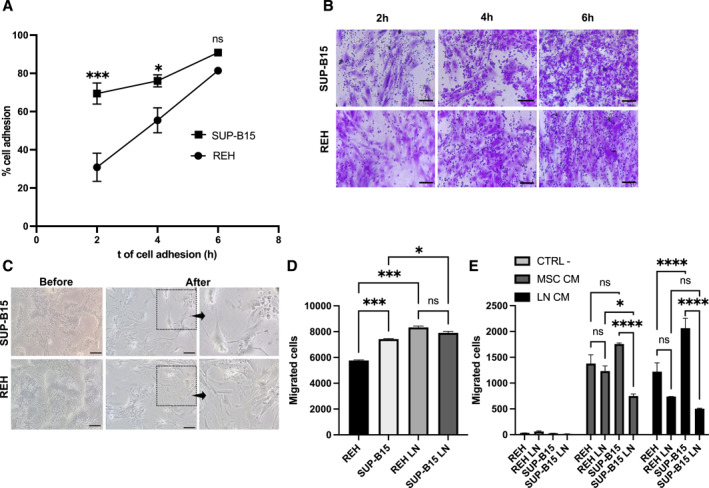
MSC modulate differentially the cell adhesion and migration capacities of SUP‐B15 and REH cells. (A) Non‐adherent cells were harvested and counted after 2, 4 and 6 h of co‐culture with MSC, determining the percentage of adherent cells. (B) Adherent SUP‐B15 and REH cells to MSC were fixed and stained with crystal violet. Microphotographs of representative experiments are shown at 20X magnification (scale bar, 100 μm). (C) SUP‐B15 and REH cells were co‐cultured with MSC for 24 h, then, leukemic cells were removed with PBS 1X and EDTA washes. Microphotographs before and after removal of leukemic cells are shown at 10X magnification (scale bar, 100 μm). Right panels, a zoomed‐in view from the boxes to show SUP‐B15 leukemic cells that remained attached to MSC. (D) For cell migration assessment, SUP‐B15 and REH cells from monocultures or LN were seeded in the upper chambers of transwell systems; the lower chambers were filled with medium containing the chemoattractant SDF‐1 (100 ng/mL). (E) Migration of leukemic cells toward MSC CM, REH LN CM or SUP‐B15 LN CM was also assessed. As negative control (CTRL ‐), cell migration toward IMDM with 0.2% BSA was used. Migrated cells were collected and counted after 24 h migration toward the lower chamber. Data are expressed as the mean ± SEM of triplicates per condition. Statistical analysis was performed using one‐way ANOVA test, followed by a Kruskal–Wallis test (A, D) and two‐way ANOVA, followed by Dunnett's post hoc test (E). No significant statistical differences (ns) and *p*‐values * < 0.05, ** < 0.01, *** < 0.001, **** < 0.0001.

The MSC survival and protective effect is based primarily on cell adherence of leukemic cells to this stromal support, a fact that has been proved extensively in diverse hematological malignancies.^6^, ^9^, ^21^, ^26^ We wonder whether this could be an initial factor to explain the different aggressiveness of these two B‐ALL subtypes. As can be seen in Figures 1A,B, more than twice SUP‐B15 cells adhere to MSC (Supporting Information Figure S1) after 2 h of co‐culture compared to REH cells. This difference shortens at 4 h and becomes nonsignificant after 6 h. Additionally, when attempts were made to detach the leukemic cells, following various protocols, it was evident that the SUP‐B15 cells remain in the wells, strongly adhered to the MSC (Figure 1C, zoom‐in view), while REH cells were completely removed (Figure 1C, zoom‐in view); because of this, additional washing procedures were necessary to completely remove all SUP‐B15 cells. Faster and stronger adhesion could give SUP‐B15 cells earlier and more effective protection by generating greater or more complete survival signals.

### 2.2 Directed migration capabilities are modulated by MSC in the aggressive SUP‐B15 cell line

REH and SUP‐B15 migrated efficiently to the chemoattractant CXCL12 (Figure 1D). Nevertheless, an increased migration was observed in SUP‐B15 cells cultured alone compared to REH cells (Figure 1D). REH and SUP‐B15 cells obtained from their corresponding LN have increased cell migration than leukemic cells cultured alone; however, in this setting there were no differences between both cell lines (Figure 1D). We also evaluated the migration capacity of leukemic cells to their own LN conditioned medium (CM) or secretome compared to control MSC CM (MSC CM). REH and SUP‐B15 cells cultured alone or obtained from each LN (REH LN and SUP‐B15 LN) did not migrate to the control medium (Figure 1E, left columns). Cell migration of both leukemic cells (culture alone or with MSC) toward their own LN CM or control CM was lower than that of toward CXCL12 (Figure 1D,E). It was also evident when comparing cells in monoculture, that both cells have equivalent migration capabilities to MSC CM (Figure 1E, middle columns), but SUP‐B15 cells migrate more than REH cells to its LN CM (Figure 1E, right columns). This situation was partially reverted when the leukemic cells came from the LN, with the REH cells migrating similar to SUP‐B15 cells (Figure 1E). Clearly, SUP‐B15 cells obtained from the LN migrated less to MSC CM and to LN CM. This is not observed in the REH cell line and agrees with the higher adherence of SUP‐B15 to MSC described above.

### 2.3 Cell proliferation decreased more over time in SUP‐B15 than in REH cells

**FIGURE 2:**
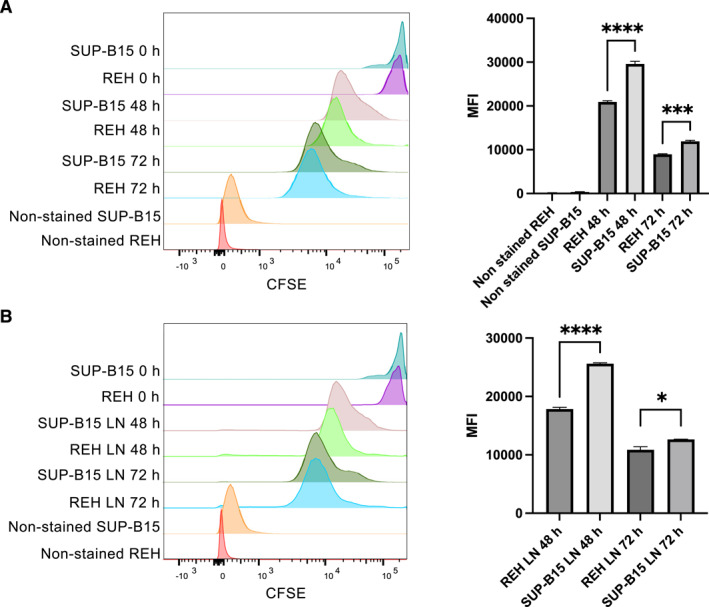
MSC induce an increase in cell proliferation of SUP‐B15 and REH cell lines. Proliferation during 48 and 72 h was assessed by flow cytometry using CFSE staining of SUP‐B15 and REH cells from (A) monocultures and (B) LN. Non‐stained cells were used as negative stain control. Freshly stained cells (0 h) were used as positive stain control. Panels on the left show one representative experiment. Panels on the right show the comparison of MFI of each condition. Data are expressed as the mean ± SEM of triplicates per condition. Statistical analysis was performed using one‐way ANOVA test, followed by a Kruskal–Wallis test. *p*‐values * < 0.05, *** < 0.001, **** < 0.0001.

Leukemic cells were labeled with CFSE and proliferation was evaluated by the loss of fluorescence after each cell division. CFSE labeling was similar in both cell lines (Figure 2A,B, *t* = 0 showed on top). Fluorescence of nonstained cells (background) was clearly differentiated from CFSE labeled cells (Figure 2A,B, bottom). In monocultures, REH cells showed greater cell proliferation than SUP‐B15 in the two time‐periods evaluated but the difference was substantially reduced after 72 h (Figure 2A). The situation was similar when leukemic cells were tested after 2 days of co‐culture with MSC, with higher proliferation of REH cells (Figure 2B). Here again, the difference in cell proliferation between REH and SUP‐B15 cells was shortened after 72 h of co‐cultures (Figure 2B). When comparing the proliferation of each cell line under the different conditions, it was observed that both REH and SUP‐B15 proliferate more in the presence of MSC, but over longer evaluation periods (72 h) this difference was completely reduced (SUP‐B15) (Supporting Information Figure S2a) or partially reverted (REH) (Supporting Information Figure S2b).

### 2.4 MSC senescence induction by SUP‐B15 and REH leukemic cells was equivalent

**FIGURE 3:**
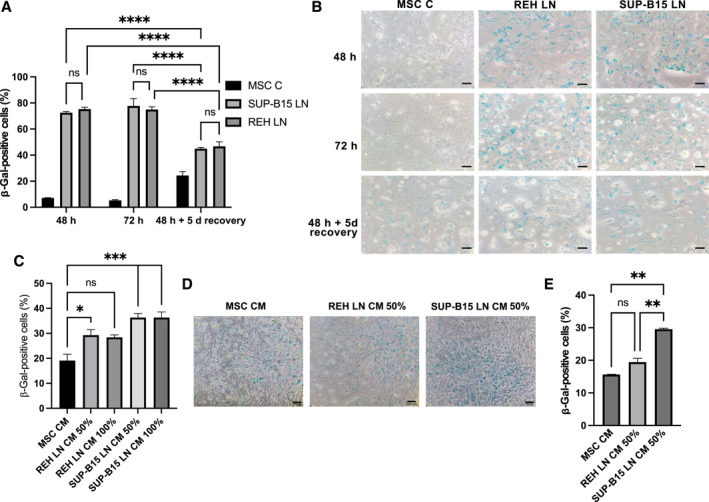
SUP‐B15 and REH cell lines increase senescence‐associated β‐galactosidase (SA‐β‐Gal) activity in MSC. (A, B) SA‐β‐Gal activity was measured in MSC after 48 and 72 h of co‐culture with REH (REH LN) or SUP‐B15 (SUP‐B15 LN) cell lines. As control, MSC were cultured alone (MSC C). Recovery from leukemic stress was also evaluated after removing SUP‐B15 and REH cells from a 48 h co‐culture, and further culturing MSC for 5 days in IMDM with 10% FBS. SA‐β‐Gal activity of MSC cultured with REH LN CM and SUP‐B15 LN CM for (C, D) 3 or (E) 9 days was also measured. As control, MSC were treated with MSC CM (C, D, and E). Microphotographs of representative experiments are shown at 10X magnification (scale bar, 100 μm). Statistical analysis was performed using two‐way ANOVA followed by Dunnett's post hoc test (A) and one‐way ANOVA test followed by a Kruskal–Wallis test (C, E). No significant statistical differences (ns) and *p*‐values * < 0.05, ** < 0.01, **** < 0.0001.

Leukemic cell adhesion to BM MSC induces in the latter morphological alterations and characteristics associated with a senescence process.^23^ Moreover, the removal of leukemic cells in an in vitro LN model reverts this process.^24^ An important issue to evaluate here was whether MSC senescence induction and its eventual reversal, after removing the leukemic cells stressor, occur or persist differentially depending on the characteristics of the leukemic cell. When evaluating morphological changes, no significant differences in cytoplasmic and nucleus areas (Supporting Information Figure S3a,b) or in cytoplasmic to nucleus ratio (C/N) were found in MSC cultured with either REH or SUP‐B15 cells (Supporting Information Figure S3c). In addition, no difference in senescence induction was observed in co‐cultured MSC with either REH or SUP‐B15 cells during 48 or 72 h, as evaluated by SA‐β‐GAL activity (Figure 3A,B). The percentage of senescent MSC increased from a basal 5% to about 70% in both cell lines. Importantly, senescence recovery, after removal of the leukemic stressor and further culturing for 5 additional days, was equivalent in both co‐cultures systems, with a decrease in SA‐β‐GAL positive cells from about 70% to near 40% (Figure 3B, right columns). It would then appear that the leukemic‐induced MSC senescence, as evaluated by morphological changes and SA‐β‐GAL activity, does not differ noticeably between these two leukemic cell lines, despite being so different in clinical prognosis.

### 2.5 MSC senescence induction by SUP‐B15 LN CM was stronger than that of REH LN CM

The LN CM also has the capacity to induce cellular senescence, as assessed by morphological alterations, SA‐β‐GAL activity and by the expression of cell cycle regulators, although to a lesser extent compared to leukemic cells.^24^ Again, no relevant differences in cytoplasmic and nucleus areas (Supporting Information Figure S3a,b) or in cytoplasmic to nucleus ratio (C/N) were found in MSC cultured with either REH LN CM or SUP‐B15 LN CM (Supporting Information Figure S3c). We next evaluated whether REH LN CM or SUP‐B15 LN CM could have a differential effect on SA‐β‐GAL activity. Both LN CM (undiluted, 100% or diluted 1:1 with medium, 50%) induced more SA‐β‐GAL positive cells compared to control MSC CM. This difference was statistically significant for the LN CM from SUP‐B15 cells (diluted or not), while it was only significant for the diluted (50%) LN CM from REH cells (Figure 3C,D). As mentioned, the amount of SA‐β‐GAL positive cells obtained by incubation with the LN CM was lower than those obtained by co‐culturing with leukemic cells (about 30% with CM vs. 70% with leukemic cells). Thus, we extended the incubation time of MSC with LN CM for 9 days with periodical re‐feeding with fresh LN CM to test if higher SA‐β‐GAL positive cells could be obtained. Although the LN CM effect was not incremental (i.e., no increased number of senescent cells was found) with this incubation period, statistically significant differences in SA‐β‐GAL activity were observed between SUP‐B15 LN CM and the REH LN CM (Figure 3E). These results suggest that MSC are more affected by the SUP‐B15 CM than by REH CM.

### 2.6 Characterization of the soluble factors present in the REH LN and the SUP‐B15 LN

**FIGURE 4:**
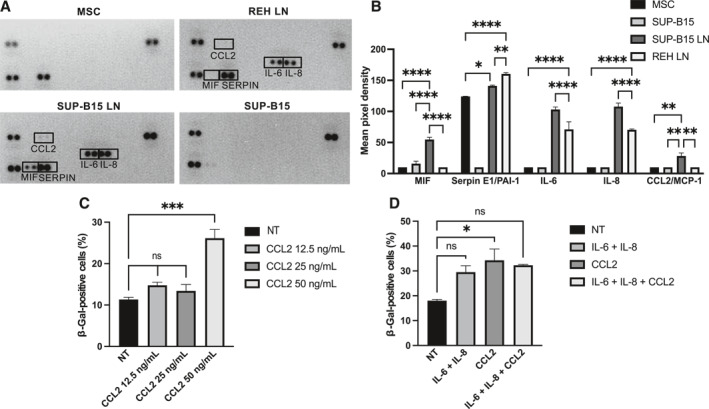
The expression of proinflammatory cytokines is substantially more upregulated in the SUP‐B15 LN than in REH LN. (A) The CM were incubated in the Proteome Profiler Human Cytokine Array; higher expressed cytokines in LN are shown (black boxes). (B) Expression of cytokines was measured by dot quantification using ImageJ software, and data are expressed as mean ± SEM. (C) MSC were incubated with CCL2 at different concentrations for 6 days, after which their SA‐β‐Gal activity was measured. (D) MSC were incubated with IL‐6 (50 ng/mL) and IL‐8 (50 ng/mL), CCL2 (50 ng/mL), or the combination of these three cytokines for 6 days, then the SA‐β‐Gal activity was measured. As controls, MSC were incubated with IMDM with 1% FBS. Statistical analysis was performed using one‐way ANOVA test, followed by a Kruskal–Wallis test (C, D) and two‐way ANOVA, followed by Dunnett's post hoc test (B). No significant statistical differences (ns) and *p*‐values * < 0.05, ** < 0.01, *** < 0.001, **** < 0.0001.

We aimed to investigate deeply the fact that SUP‐B15 LN CM induced a stronger MSC senescence and the possible functional consequences of this. The set of soluble factors secreted by senescent cells is called the senescence‐associated secretory phenotype (SASP) and has been shown to be a complex mixture of cytokines and growth factors, whose composition depends on the trigger, the time of stimulation, the cell type, and the cellular context.^27^ For this reason, we determined its composition using a microarray assay identifying 36 different soluble factors. We had previous information on the REH cells and REH LN secretomes,^11^ and therefore the four tests available were used for the MSC control, SUP‐B15 LN CM and REH LN CM, and the SUP‐B15 cell line alone. As shown (Figure 4A,B), IL‐6 and IL‐8 were significantly increased in both REH LN and SUP‐B15 LN compared to control MSC, with the SUP‐B15 LN showing an increased expression (about 50%) of both cytokines, compared to REH LN. Two other cytokines were increased CCL2/MCP1 (2 times) and MIF (4 times) only in the SUP‐B15 LN when compared to control MSC. This differential expression was due to the interaction between MSC and leukemic cells, since SUP‐B15 or MSC alone do not secrete these cytokines. Only Serpin E1/PAI‐1 was highly expressed in control MSC and in both LN. Our interest was then focused on those increased cytokines in the SUP‐B15 LN that could be relevant for the dynamic interaction of leukemic cells and MSC, that is, IL‐6, IL‐8 and CCL2.

### 2.7 Specific cytokines simulated the LN CM effect on MSC senescence induction

Next, we evaluated the effect of recombinant human cytokines on MSC senescence induction. IL‐6 and IL‐8 were used at 50 ng/mL^28^ and several concentrations of CCL2 were first tested for senescence induction in MSC (Figure 4C). No important and significant increase in SA‐β‐GAL positive cells was observed with 12.5 ng/mL or 25 ng/mL CCL2, whereas a significant 2.5‐fold increase in SA‐β‐GAL positive cells was observed with 50 ng/mL CCL2 stimulation of MSC (Figure 4C). A mixture of IL‐6 and IL‐8 (both at 50 ng/mL) or IL‐6, IL‐8 and CCL2 (all at 50 ng/mL) and CCL2 alone (50 ng/mL) induced MSC senescence, but only the difference between control cells and those treated only with CCL2 was statistically significant (Figure 4D). Of note, we did not observe a synergistic effect of stimulating MSC with IL‐6/IL‐8 and CCL2 (Figure 4D, right column).

### 2.8 No changes in sensitivity to MTX, VCR or DOX treatments of REH or SUP‐B15 cells were observed with CCL2‐pretreated MSC

It has been shown that MSC senescence has a modulatory role in drug resistance in various hematological malignancies.^24^, ^29^, ^30^ Thus, our aim was to reveal if MSC senescence induction by pre‐treatment with CCL2 could alter differentially the sensitivity of leukemic cells to the different cytotoxic drugs used in B‐ALL patients. For defining the drugs and the concentrations to be evaluated here, we looked for the reported drug IC_50_ in the REH and SUP‐B15 cell lines, or from our own dose‐response experiments, when no information was available (Supporting Information Figure S4a). Two drug concentrations were then selected, the IC_50_ and the corresponding double concentration (Supporting Information Table S1).

**FIGURE 5:**
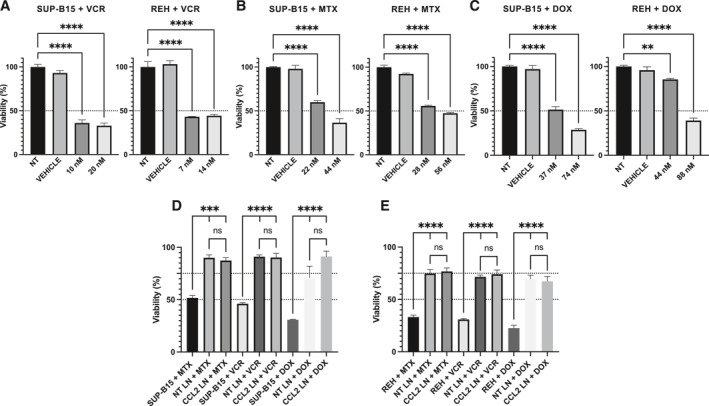
CCL2 pre‐treatment does not alter the capacity of MSC to protect SUP‐B15 and REH cell lines against drug treatment. MTT assay was used to assess cell viability. SUP‐B15 and REH cell lines were treated with the IC_50_ and the double concentration of the IC_50_ of (A) vincristine (VCR), (B) methotrexate (MTX) and (C) doxorubicin (DOX), as indicated. As controls, non‐treated (NT) cells and vehicle‐treated cells were used. (D) MSC were pre‐treated with CCL2 (CCL2 LN; 50 ng/mL) or IMDM with 1% FBS (NT LN) for 48 h followed by co‐culture with SUP‐B15 or (E) REH cell lines, then MTX, VCR or DOX treatments were added at two times the IC_50_ of each drug. Dot lines indicate 50% (A–E) and 75% viability (D, E). Data are expressed as the mean ± SEM of triplicates per condition. Statistical analysis was performed using one‐way ANOVA test, followed by a Kruskal–Wallis test. No significant statistical differences (ns) and *p*‐values ** < 0.01, *** < 0.001, **** < 0.0001.

From the six cytotoxic drugs tested, only three (vincristine, VCR; methotrexate, MTX; and doxorubicin, DOX) showed cytotoxicity near or higher than 50% for the SUP‐B15 and REH leukemic cell lines at least at one of the two concentrations evaluated (Figure 5A–5C). The other three drugs were not (prednisolone and dexamethasone) or slightly (asparaginase) cytotoxic for the REH cell line at the concentrations used here (Supporting Information Figure S4b–d), and were therefore not chosen for comparison. The vehicle DMSO at different concentrations used to dissolve the drugs did not alter the viability of REH or SUP‐B15 cell lines (Figure 5A–5C). We also checked that MTX, VCR, or DOX treatments (Supporting Information Figure S5a) and CCL2 pre‐incubation did not affect MSC viability or cell proliferation (Supporting Information Figure S5b). As can be seen and as expected, MSC efficiently protects REH and SUP‐B15 cells from drug treatments (Figure 5D,E), increasing leukemic cell viability from about 25% to 30% to 70%–80% in the REH cell line for MTX, VCR, and DOX treatments; and from about 30% to 45% to almost 90% in the SUP‐B15 cell line for MTX, VCR, and DOX treatments. Of note, this protection was not altered by the pre‐treatment of MSC with 50 ng/mL CCL2 for 48 h (Figure 5D,E), a treatment inducing about 30% senescence in MSC.

Finally, we also verified that drug protection in the co‐cultures was not due to either (1) an effect of the secreted cytokines directly on leukemic cells or (2) an indirect effect caused by absorption of the drugs by MSC and hence less drug bioavailability in the wells. Incubation of REH (Supporting Information Figure S5c) or SUP‐B15 (Supporting Information Figure S5d) cells, with the CM from the corresponding LN did not protect the leukemic cells from MTX, VCR or DOX treatments, and cytotoxicity was almost equivalent to control medium. Only with REH cells a small protection effect to MTX was observed when control medium was compared to MSC CM, but no differences were observed between LN CM and controls (Supporting Information Figure S5d). In the case of REH cells treated with DOX, it was observed that the MSC CM or the LN CM, instead of protecting the leukemic cells, induced, on the contrary, a slight increase in the cytotoxicity of the compound (Supporting Information Figure S5c). Therefore, the protection of leukemic cells observed in the co‐cultures with MSC is not due to a direct effect of the soluble factors present in the LN on leukemic cells. For testing drug bioavailability after incubation with cells, MSC were incubated with MTX, VCR or DOX for 48 h and the supernatants of these incubations were used for testing their cytotoxicity toward SUP‐B15 (Supporting Information Figure S6a) and REH (Supporting Information Figure S6b) cell lines and compared to freshly prepared drugs. There were minimal differences between the freshly prepared drugs and the supernatant of MSC pre‐incubated with drugs (Supporting Information Figure S6a,b). We also found that the drugs used do not lose cytotoxicity when incubated alone at 37^o^C for 48 h (Supporting Information Figure S6a,b). From these results, it is clear that protection from MTX, VCR and DOX is based solely on a specific role of MSC.

### 2.9 The aggressive SUP‐B15 cell line alters MSC stemness functions

**FIGURE 6:**
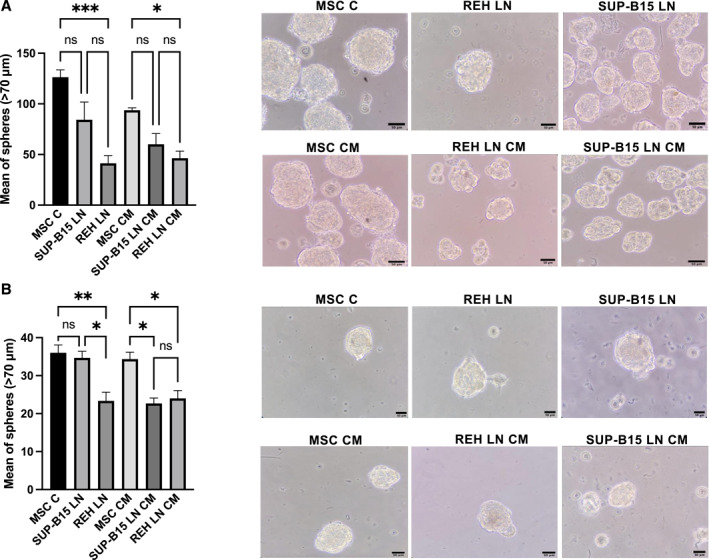
MSC self‐renewal is decreased by REH but not by SUP‐B15 cell line. Self renewal was assessed using the sphere‐forming assay. (A) MSC were first co‐cultured with SUP‐B15 (SUP‐B15 LN) or REH (REH LN) cell lines, or treated with SUP‐B15 LN CM or REH LN CM for 72 h. As controls, MSC were cultured alone (MSC C) or treated with MSC CM. Then, MSC were collected and plated in ultra‐low adherence microplates with sphere inducing medium for 5 days. Mesenspheres were measured and counted using an inverted microscope. (B) Mesenspheres were then collected, disaggregated and plated again in sphere inducing medium and further cultured for 5 days. Bars (left panels) show the counts of those mesenspheres with a diameter greater than 70 μm. Representative microphotographs (right panels) are shown at 40X magnification (scale bar, 50 μm). Data are expressed as the mean ± SEM of triplicates per condition. Statistical analysis was performed using one‐way ANOVA test, followed by a Kruskal–Wallis test. No significant statistical differences (ns) and *p*‐values * < 0.05, ** < 0.01, *** < 0.001.

We proceeded to evaluate if the two leukemic cell lines differentially affected MSC self‐renewal, as assessed by the sphere‐forming assay.^31^ As can be seen in Figure 6A, both leukemic cell lines reduced the number of mesenspheres formed by MSC, but only those co‐cultured with the REH cell line showed significant differences when compared to control MSC. Of relevance, a second‐generation sphere‐forming assay (disaggregating the cells within spheres by enzymatic treatment and re‐inducing spheres formation) showed that MSC from the SUP‐B15 LN had totally recovered its self‐renewal capacity, showing no difference with the control MSC (Figure 6B). On the contrary, MSC from the REH LN remained low and now presented statistically significant differences with the MSC SUP‐B15 LN (Figure 6B). This showed that SUP‐B15 cells better preserved the self‐renewal function of MSC. The situation was similar when the sphere assay was performed with LN CM, with the REH LN CM affecting more self‐renewal (Figure 6A). Nevertheless, the second‐generation sphere‐forming assay showed no differences between REH and SUP‐B15 LN CM (Figure 6B). This points to the relevance of cell‐to‐cell contact in altering this functional property of MSC.

**FIGURE 7:**
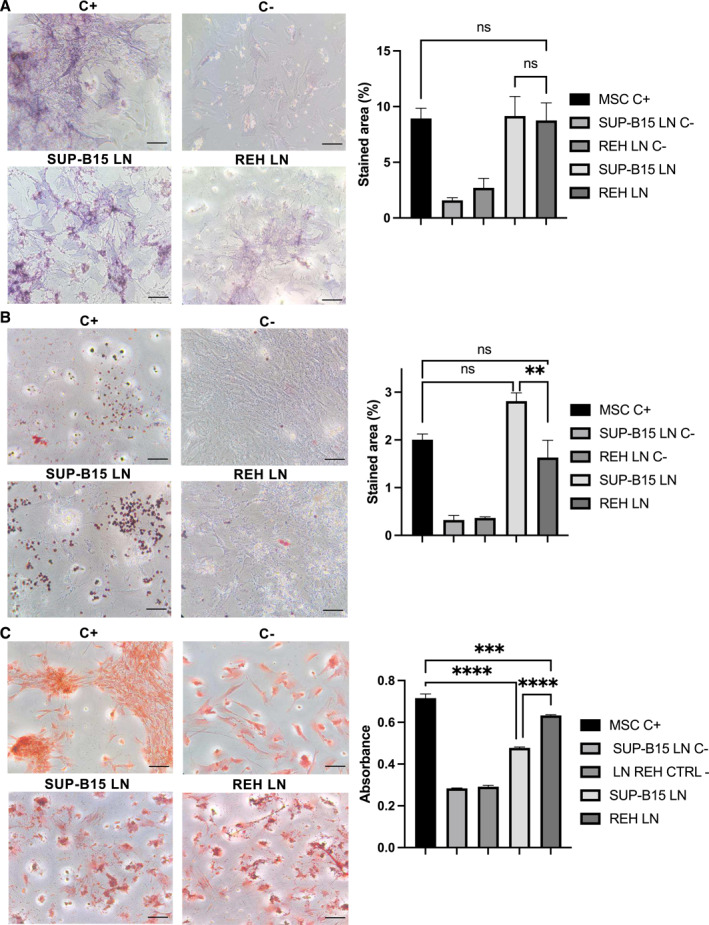
SUP‐B15 cell line strongly alters the multipotent differentiation capacity of MSC. MSC were co‐cultured with SUP‐B15 or REH cell lines for 72 h, after which leukemic cells were removed and induction media for osteogenic, adipogenic and chondrogenic differentiation was added. Cultures were maintained for 14 days for osteogenic and adipogenic lineages and 21 days for chondrogenic lineage. (A) Staining with NBT/BCIP was used for osteogenic differentiation; (B) Oil Red for lipid vacuoles in adipogenic differentiation and (C) safranin O for chondrogenic differentiation. Stained area for osteogenic and adipogenic cultures were determined using Image J software (a, b right panels). Safranin O staining of chondrogenic cultures was solubilized using DMSO and absorbance at 550 nm was measured (C right panel). MSC cultured alone followed by induction of differentiation were used as positive control (C+). MSC cultured in IMDM with 10% FBS instead of differentiation induction media were used as negative control (C‐). Representative microphotographs (left panels) are shown at 10X magnification (scale bar, 100 μm). Data are expressed as the mean ± SEM of triplicates per condition. Statistical analysis was performed using one‐way ANOVA test, followed by a Kruskal–Wallis test. No significant statistical differences (ns) and *p*‐values ** < 0.01, *** < 0.001, **** < 0.0001.

We also assessed whether the multipotent differentiation (MPD) capacity of MSC was differentially affected by each of the leukemic cell lines. No differences were observed in MSC osteogenic differentiation between REH and SUB‐15 co‐cultures (Figure 7A). In contrast, the SUP‐B15 leukemic cells induced more adipogenic (Figure 7B) and less chondrogenic (Figure 7C) differentiation of MSC than the REH leukemic cells. We have also evaluated the effect of REH LN CM and SUP‐B15 LN CM on MPD capacity. No statistically significant differences were found between REH and SUP‐B15 LN CM, although the trend showed above employing whole cells was maintained (Supporting Information Figure S7a–c). Therefore, REH and SUP‐B15 leukemic cells affect differentially MDP, with the direct contact between leukemic cells and MSC having a central role.

### 2.10 Adipogenic‐induced MSC showed greater protection against cytotoxic drugs

The purpose now was to determine whether these changes in stem cell function, particularly in MPD capacity, could give leukemic cells a survival advantage, which would eventually be favorable for the most aggressive ones. To test this, we first have established a stromal cell support with variable amounts (0, 50% and 100%) of MSC induced to adipogenic differentiation (AD) for 7 or 14 days (see materials and methods section). As can be seen, a 7‐day adipogenic induction produces a uniform stromal support with MSC and pre‐adipocytes homogenously distributed in the wells (Supporting Information Figure S8a). This was not the case with the 14 days adipogenic induction period of MSC (Supporting Information Figure S8b). Therefore, we have used a 7‐day adipogenic induction period to test the capacity of these different stromal supports to sensitize leukemic cells to drug treatment.

**FIGURE 8:**
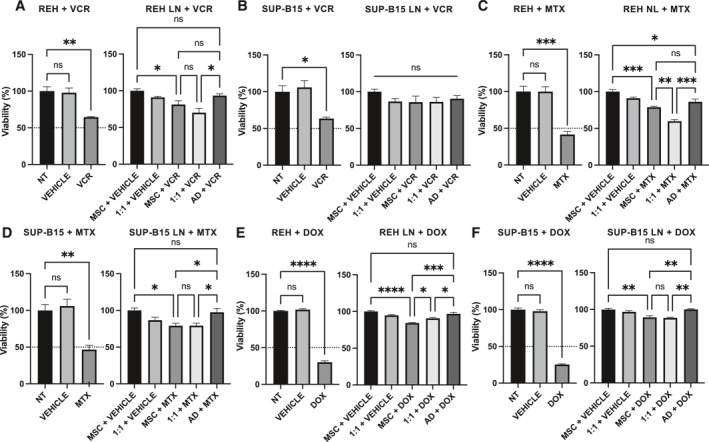
Adipogenic differentiated MSC show greater protection toward drug treatments in SUP‐B15 and REH cell lines. MTT assay was used to assess cell viability. For LN, three types of stromal support were established: MSC, adipogenic‐induced MSC for 7 days (AD) and a mixture 1:1 of MSC and AD (1:1). Then, these were co‐cultured with SUP‐B15 or REH cell lines followed by treatment with VCR (A, B), MTX (C, D) and DOX (E, F). Left panels show simultaneous drug treatments of SUP‐B15 or REH cell lines alone for comparison. Right panels show drug treatments of LN. Non‐treated (NT) cells and vehicle‐treated cells were used as controls. Data are expressed as the mean ± SEM of triplicates per condition. Statistical analysis was performed using one‐way ANOVA test, followed by a Kruskal–Wallis test. No significant statistical differences (ns) and *p*‐values * < 0.05, ** < 0.01, *** < 0.001, **** < 0.0001.

Protection toward cytotoxic drugs (VCR, MTX and DOX) was obtained with the three stromal support settings (only MSC, 1:1 AD:MSC, and only AD) (Figure 8A–F, please compare panels on the left with panels on the right). Nevertheless, a statistically significant difference in the susceptibility to drug treatment was observed when the stromal support was prepared with only AD‐induced MSC compared to the 1:1 mixture or MSC alone. This was the case for REH cells treated with VCR (Figure 8A), MTX (Figure 8C) and DOX (Figure 8E) and for SUP‐B15 cells treated with MTX (Figure 8D) and DOX (Figure 8F). Protection to VCR treatment in the SUP‐B15 cell line was similar in the three stromal conditions (Figure 8B). Of note, the difference between a stromal support with MSC alone and with 100% AD‐induced MSC was significantly different for the SUP‐B15 cell line with MTX treatment (Figure 8D) and for REH and SUP‐B15 cell lines with DOX treatment (Figure 8E,F). As with MSC, there was no loss of drug cytotoxic capacity when these were pre‐incubated with MSC induced to AD, showing that drugs are not adsorbed by adipocytes (Supporting Information Figure S6c,d). On the contrary, in the SUP‐B15 cells and only with VCR, a slight increase in cytotoxicity was found when the drug was pre‐incubated alone or with adipocytes (Supporting Information Figure 6c,d). These results show that SUP‐B15 cells are better protected toward VCR, MTX, and DOX in all settings, with stronger protection in the AD‐induced cell differentiation. Therefore, SUP‐B15 cells, with a greater capacity to induce AD in MSC, would be better protected than REH cells against these drugs.

## 3 DISCUSSION

The survival capacity of patient‐derived primary B‐ALL cells on MSC in vitro has been shown to be a consistent predictor of disease aggressiveness and treatment outcome,^32^, ^33^ suggesting that the interaction between MSC and B‐ALL cells differentially defines and modulates properties of leukemic cells that make them more or less aggressive. We have here established a LN by incubating MSC with two leukemic cell lines having different genetic abnormalities and opposing prognosis, good for REH and bad for SUP‐B15 cells. We have shown here that the aggressiveness of a particular leukemia subtype involves both intrinsic aspects of the leukemic cells, revealed by their particular interaction with MSC, and specific alterations of MSC in the LN, which together impact on the performance and functional capabilities of the leukemic cells. Umbilical cord blood MSC were selected here not only for the ease of their collection but also for their good proliferation capacity, similar biological characteristic to BM MSC and to avoid the heterogeneity and infrequency of the latter,^34^, ^35^ allowing the establishment of richer and more homogeneous stromal support.

First, we have shown that adherence to MSC of the SUP‐B15 cell line is stronger and faster than that of the REH cell line. This simple fact would give SUP‐B15 leukemic cells the possibility of receiving MSC survival signals in a timely manner. In turn, stronger cell adhesion to MSC would imply that adhesion molecules‐derived intracellular signals are more complete or diverse and would also help to select those leukemic cells that are more resistant to treatment with cytotoxic drugs.^36^ These differences in adherence could be related to the osteopontin‐mediated adhesion mechanism in MSC, present in the SUP‐B15 cell line and not in the REH cell line, which was recently described.^37^ Indeed, the authors suggested that specific genetic alterations might influence the disease niche. Differential up‐regulation of cell adhesion molecules and cell adherence offers the possibility of blocking the specific surface players or intracellular signals responsible for this cell interaction.^14^, ^23^, ^38^, ^39^, ^40^

Additionally, SUP‐B15 cells showed greater cell migration than REH cells toward both CXCL12 and the corresponding LN CM. This finding is not trivial either, since it has been shown in xenograft mouse models that leukemic cells having increased motility were able to engraft in the BM more rapidly than the slower counterpart^41^ and were able to survive therapy better than those with less motility.^42^ Increased motility and migration would allow spread of leukemic cells and the colonization of new sites in the BM, favored precisely by these enhanced properties of SUP‐B15 cells. Of note, MSC isolated from B‐ALL patients retained, over several passages in vitro, the cell reprogramming mechanisms responsible for this increased migration.^41^ This would ensure a prolonged and ideal microenvironment for effective competition of leukemic cells.^9^ It was proposed that therapeutic interventions should target the migration and promiscuous interactions with the surrounding microenvironment in order to combat the survival of chemoresistant ALL cells.^43^ Regarding cell proliferation, there was an initial period (before 48 h) in which SUP‐B15 cells showed reduced cell proliferation compared to REH cells, but this difference became much less noticeable 24 h later. This would suggest the existence of two temporal stages, an initial one in which SUP‐B15 leukemic cells promptly adhere to MSC, showing lower cell proliferation; and a later one, with SUP‐B15 cells increasing cell proliferation and migration.

Leukemic cells induced important alterations in MSC including a senescent process that has been demonstrated both in *vitro* co‐cultures and in MSC isolated from patients with different hematological neoplasm.^23^, ^24^, ^29^, ^44^, ^45^ In B‐ALL, this process was initiated by cytoplasmic and mitochondrial ROS production^23^, ^24^ and was maintained and expanded by the paracrine effect of secreted soluble factors, having important implications for disease progression. MSC senescence and its SASP modify directly or through a paracrine effect different components of the niche microenvironment,^46^ affecting the susceptibility to drug treatment of primary B‐ALL cells.^24^ For these reasons, we have investigated if there would be differences in MSC senescence induction by REH and SUP‐B15 cells. We found no differences in the early induction of senescence (48 and 72 h of co‐culture) between the two cell lines, and we saw no variations in recovery from senescence once the leukemic stress was removed. This ability of MSC to recover from senescence and ROS effects, in the absence of leukemic stress, shows their efficient repair capacity^47^ and would suggest that therapeutic strategies that inhibit the induction of senescence or senescence itself (senolytics, senomorphs, or anti‐adherent antibodies) could be a complementary and effective therapy for all subtypes of B‐ALL.^48^ In fact, the absence of MSC genotoxic stress was associated with a lower risk for leukemia progression.^10^ Additionally, partial functional restoration of BM mesenchymal stroma would in turn allow the normalization of hematopoiesis.

SASP is responsible in part of the senescence effects in the tumor microenvironment,^49^, ^50^ and transient exposure to the SASP can promote cell plasticity and tissue regeneration of cancer cells.^51^ We initially studied LN CM (“SASP”) effects in MSC senescence induction and then its soluble factors composition. First, and in contrast to what we found with leukemic cells, the SUP‐B15 LN CM, but not the REH LN CM, showed an increased MSC senescence that was significantly different to control MSC CM. Furthermore, by extending the incubation time of MSC with the LN CM for 9 days, a significant difference in MSC SA‐β‐GAL activity was found between the SUP‐B15 LN CM and the REH LN CM. This suggested a greater capacity of SUP‐B15 LN CM to induce MSC senescence compared to REH LN CM, and therefore a different composition of soluble factors, which we assessed by a microarray assay. In fact, several cytokines (CCL2, IL‐6, IL‐8 and MIF) were found in greater amounts in the SUP‐B15 LN CM. Noteworthy, patients with hematological malignancies have increased serum levels of CCL2, IL‐6 and IL‐8 that have been associated with symptoms and with a poor prognosis.^52^, ^53^, ^54^, ^55^ The augmented secretion of these cytokines by the SUP‐B15 leukemic cells could then partly explain their increased aggressiveness.

Interestingly, although the LN CM and individual cytokines or different combinations induced senescence in MSC, only the CCL2 stimulation of MSC for 3 days induced a number of senescent MSC that was significantly different from control MSC. We then proceeded to evaluate the role of MSC CCL2 pre‐incubation in drug susceptibility in both REH and SUP‐B15 B‐ALL cell lines. CCL2‐pretreated MSC were almost not affected in their capacity to protect any of the leukemic cell lines with the three drugs tested (VCR MTX and DOX), even though CCL2 treatment induced MSC senescence. This contrasts with what we found with BM‐MSC and primary leukemic cells,^24^ difference that can be attributed to the cells used in each case (BM vs. UCB and primary leukemic cells vs. leukemic cell lines). It should also be mentioned that the other three drugs (prednisolone, dexamethasone, and asparaginase) not evaluated here in the MSC pre‐incubation assays with CCL2 or LN CM, had, unexpectedly, less cytotoxic effect in the less aggressive REH cell line. This, together with our results of the pre‐treatment of MSC with CCL2 or the CM, would indicate that the aggressiveness of the cells is not related to their inherent resistance to certain drugs, per se, but probably to the fact that leukemic cells interact in a particular way with stromal cells^56^ selecting for more resistant subclones, something that merits further investigation.

Alterations in stemness features could also be responsible of diminishing leukemic cell fitness and/or increasing susceptibility to drug treatment.^22^ The issue now was to disclose whether the senescence process induced by leukemic cells disturbs stemness functions, and in turn, how these changes affect leukemia survival or performance. We have shown here, that the two cell lines differentially affected MSC self‐renewal, with the SUP‐B15 cell line preserving it better. Comparable results were obtained when self‐renewal was evaluated with LN CM, that is, SUP‐B15 LN CM inducing more spheres than REH LN CM. Importantly, a second‐generation sphere assay showed that in MSC from the SUP‐B15 co‐cultures self‐renewal capacity was almost identical to control cells, while in MSC from REH co‐cultures the deficiency persisted. One might assume, that maintaining this highly relevant function, not only for MSC homeostasis and regeneration but also for giving support to BM cells, is essential for leukemic cells growth and maintenance.^22^ As REH cells affect more MSC self‐renewal their survival is more compromised.

Next, we have studied the effect that REH and SUP‐B15 leukemic cells have on MSC MPD capacity. MSC osteogenic differentiation has been in general more controversial, with groups reporting reduced^24^ and other normal capacity.^57^ Nevertheless, osteoblasts were reduced in BM of children with B‐ALL at diagnosis^58^ with B‐ALL cells playing a main role.^59^ In our system, no differences between REH and SUP‐B15 leukemic cells and the control were observed in osteogenic differentiation. On the other hand, SUP‐B15 cells induced in MSC greater AD and less chondrogenic differentiation than REH cells. Since adipocytes comprise about 45% of total BM regions where hematopoiesis is active^60^ and have a shielding effect for chemotherapy exposure in acute leukemia,^61^, ^62^ we have established stromal supports consisting of solely MSC, a mixture 1:1 of MSC and AD‐induced MSC, and only AD‐induced MSC. Interestingly, we showed here that MSC induced to AD protect REH cells better from VCR, MTX, or DOX treatments and SUP‐B15 cells from MTX and DOX treatments. These results suggest that the aggressive SUP‐B15 cells, with the capacity of inducing higher AD, would be better shielded from the cytotoxic effect of these compounds. It has been shown that adipocytes contribute to chemoresistance by metabolic changes in leukemic cells^63^ or by adsorption and further metabolism of drugs.^64^ We have shown here that none of the drugs used here were absorbed by adipocytes, suggesting that protection was related to MSC intrinsic mechanisms.

All in all, we have shown here that the genetic characteristics of particular B‐ALL subtypes impact differentially the stromal microenvironment, conferring or revealing specific functions (cell adherence and migration) that favor leukemic cell survival. These leukemic cells affect differently the secretome and stem cell functions of MSC. In particular, the aggressive SUP‐B15 cells preserve better MSC self‐renewal and hence the stability of a functional LN favorable for leukemic cell survival. This is a relevant point, as it is a counterintuitive argument, since it is expected that the most aggressive cells are those that cause the most damage to the BM microenvironment. It would be interesting to evaluate this issue in in vivo models or in BM samples from B‐ALL patients, an obvious limitation of our in vitro model. Likewise, we have shown that SUP‐B15 cells induce a greater AD capacity of MSC favoring drug resistance. Here again, if this knowledge can be extended to in vivo models or to samples from patients with B‐ALL, it will eventually allow the development of new strategies to increase the efficacy of microenvironment‐targeted therapies.

## 4 MATERIALS AND METHODS

### 4.1 MSC isolation and characterization

MSC were obtained from umbilical cord blood samples (*n* = 2) from two healthy donors in labor after they gave their approval by signing the informed consent from the “Instituto Distrital de Ciencia Biotecnología e Innovación en Salud,” Bogotá (IDCBIS). The Ethical Committee of the Faculty of Medicine, Universidad Nacional de Colombia, approved the protocols for isolation and characterization of MSC. Ficoll‐Hypaque density gradient centrifugation (Histopaque, d = 1.077 g/mL, Sigma‐Aldrich, St. Louis, MO, USA) was used to isolate mononuclear cells (MNC), then, MNC were plated in 75 cm^2^ cell culture flasks (SPL Life Sciences, Pocheon, South Korea) in Iscove's Modified Dulbecco's Medium (IMDM, GIBCO, Thermo Fisher Scientific, New York, NY, USA) supplemented with 1% nonessential amino acids, 1% sodium pyruvate (LONZA, Walkersville, MD, USA) and 10% fetal bovine serum (FBS, GIBCO‐Life Technologies, New York, NY, USA). Cultures were maintained at 37°C, 5% CO_2_ and a humidified atmosphere for 48 h. Under these conditions MSC were cultured until reaching a confluence of >80% and used in passages 2‐5 in different experiments.

For immunophenotypic characterization of MSC, cells were labeled and analyzed using a FACSAria IIIup flow cytometer (Becton Dickinson Biosciences, San Jose, CA, USA) with the following monoclonal antibodies: FITC mouse antihuman CD73 (clone AD2, BD Pharmingen, San Jose, CA, USA), APC mouse antihuman CD105 (clone 266, BD Pharmingen, San Jose, CA, USA), FITC mouse antihuman CD90 (clone 5E10, Biolegend, San Diego, CA, USA), FITC antihuman CD44 (clone G44‐26, BD Pharmingen, San Jose, CA, USA), anti‐human CD34 (clone REA1164, Miltenyi Biotec, Bergisch Gladbach, Germany), and antihuman CD45 (clone 2D1 BD Pharmingen, San Jose, CA, USA). For data analysis the FACSDiva and FlowJo software (Becton Dickinson Biosciences, Sunnyvale, CA, USA) was used. Multipotent differentiation capacity of MSC toward osteogenic, adipogenic, and chondrogenic lineages was evaluated using specific induction, staining, and analysis protocols for each lineage. Osteogenic differentiation was achieved after cultures were maintained for two weeks at 37ºC and 5% CO_2_ in MEMα medium supplemented with 10% FBS, 100 nM dexamethasone, 10 mM β‐glycerophosphate and 200 μM ascorbic‐2‐phosphate (all reagents from Sigma‐Aldrich, St. Louis, MO, USA). For adipogenic differentiation, cells were cultured for 1–2 weeks in MEMα medium supplemented with MEMα medium supplemented with 10% FBS, 1 mM dexamethasone, 200 μM indomethacin, and 10 μg/mL insulin (all reagents were from Sigma‐Aldrich, St. Louis, MO, USA) and 500 μM isobutylmethylxanthine (Abcam, Cambridge, MA, USA). For chondrogenic differentiation, cultures were maintained for three weeks at 37ºC and 5% CO_2_ in MEMα medium supplemented with 1% FBS, 10 ng/mL TGFβ‐1, 1 μM ascorbic acid, 1 μM dexamethasone (all reagents from Sigma‐Aldrich, St. Louis, MO, USA), 1% insulin‐transferrin‐selenium, and 1% sodium pyruvate. After the differentiation periods, cultures were fixed with 10% formaldehyde at room temperature (RT) and washed once with PBS 1×. For staining, 1‐Step™ NBT/BCIP Substrate Solution (ThermoFisher Scientific, Grand Island, NY, USA) was added to osteoblasts and incubated at RT for 15 min in the dark. Adipocytes were stained using 0.35% Oil Red O solution (Sigma‐Aldrich, St. Louis, MO, USA) at RT for 50 min. Staining of chondrocytes was performed with 0.1% Safranin O (Sigma‐Aldrich, St. Louis, MO, USA) at RT for 15 min; then, the staining solution was removed, and cultures were washed with PBS 1×. Microphotographs were taken using an inverted microscope.

### 4.2 Leukemia cell lines

The human B‐ALL cell lines REH (ATCC CRL‐8286) and SUP‐B15 (ATCC CRL‐1929) were cultured at 37ºC, 5% CO_2_ and a humidified atmosphere in RPMI 1640 medium (GIBCO, ThermoFisher Scientific, Grand Island, NY, USA) or IMDM medium, respectively, both supplemented with 10% FBS.

### 4.3 Establishment of LN with leukemic cell lines, MSC and/or adipocytes

Upon reaching a confluence of >90%, MSC were trypsinized, collected and seeded at a confluence of near 70%, depending on the experiment. Then, REH or SUP‐B15 cells were added to the MSC monolayer at a ratio of 1:5 or 1:7 (MSC:B‐ALL). The co‐cultures were maintained for 1–3 days at 37ºC and 5% CO_2_ in a humidified atmosphere. For the LN established with adipocytes, MSC were seeded and induced to adipogenic differentiation for 7 days as described below. If the stromal cell support was composed of adipocytes (50%) and noninduced MSC (50%), the latter were seeded on top of a 7‐day adipogenic induced culture and further incubated for 2 days. Then, REH or SUP‐B15 cells were added as described. When required, leukemic cells were removed by washing four times the culture with cold PBS 1× and another wash with PBS 1× + EDTA.

### 4.4 Condition medium (CM) preparation

5 × 10^5^ MSC were seeded in 75 cm^2^ cell culture flasks and cultured until they reached a confluence of 80%. Then, medium was removed, and the culture was washed once with incomplete IMDM medium. Subsequently, 2.5 × 10^6^ REH cells or 3.5 × 10^6^ SUP‐B15 cells were seeded on top of the MSC in IMDM medium with 1% FBS. After 24 h of incubation at 37ºC and 5% CO_2_, the medium was collected and centrifuged at 500 × *g* for 5 min. The supernatant was collected, filtered through a 0.2 μm pore syringe filter (Corning Life Sciences, Oneonta, NY, USA), aliquoted and stored at −20ºC for later experiments.

### 4.5 Cell adhesion assay of leukemic cells to MSC

4 × 10^4^ leukemic cells of either REH or SUP‐B15 cell lines were added to 8 × 10^3^ MSC previously seeded in 96‐well microplates. Functional leukemic cell adhesion was assessed within the first 2, 4, and 6 h of incubation. Cultures were washed using PBS 1×, loosely attached cells were collected and counted using a Neubauer chamber. Subsequently, cultures were stained with crystal violet (0.5%) and a photographic register was taken.

### 4.6 Leukemic cells migration evaluation

Transwell systems were used to assess the migration capacity of REH and SUP‐B15. Leukemic cells were previously cultured alone or with MSC for 24 h as previously described. Then, cultures were washed with PBS 1× to detach leukemic cells. These were collected, washed and resuspended in migration buffer (IMDM medium with 0.2% BSA (GIBCO‐Invitrogen, Grand Island, NY, USA)). Afterward, 7 × 10^4^ REH or SUP‐B15 cells were seeded in the upper chamber of the insert (5 μm pore size, 6.5 mm diameter, Corning Incorporated, Corning, NY, USA). The lower chamber was filled with different chemoattractants including MSC CM, LN REH CM or LN SUP‐B15 CM, migration buffer (negative control) and CXCL12 100 ng/mL (MyBioSource, San Diego, CA, USA) as a positive control. After 24 h of incubation at 37ºC and 5% CO_2_ in a humidified atmosphere, cells in the lower chamber were counted using a Neubauer chamber.

### 4.7 Cell proliferation assay of leukemic cell lines

Leukemia cell proliferation was assessed by cell staining with carboxyfluorescein diacetate succinimidyl ester (CFSE, CellTrace™ CFSE Cell Proliferation Kit, Invitrogen, Eugene, OR, USA). REH or SUP‐B15 cells were washed and resuspended in PBS 1× with 0.1% BSA and incubated with 5 μM CFSE at 37ºC for 7 min. Afterward, cells were resuspended in complete IMDM medium, incubated on ice for 5 min, and washed three times at 400 × *g* for 7 min. Upon the last centrifugation, cells were resuspended in complete IMDM medium and seeded on MSC or cultured alone, and incubated at 37ºC for 48 or 72 h. Finally, leukemia cells were detached by PBS 1× washes and collected. Then, cells were washed twice in PBS 1× at 400× *g* for 5 min. Proliferation of CFSE‐stained cells was evaluated by flow cytometry measuring the mean fluorescence intensity (MFI). For negative control, nonstained cells were used, and freshly stained cells were used as positive control.

### 4.8 Mesensphere formation assay

Prior to the mesensphere formation assay, MSC cells were stained using CFSE, as described above. Then, MSC were either co‐cultured with REH or SUP‐B15 cells, or treated with LN REH LN CM or SUP‐B15 LN CM for 72 h at 37ºC and 5% CO_2_, a re‐feeding of medium was done at 36 h. Meanwhile, sphere‐inducing medium was prepared in Dulbecco's modified Eagle's medium (DMEM)/F12 (1:1)/human endothelial (1:2) serum‐free medium (GIBCO, ThermoFisher Scientific, Grand Island, NY, USA) supplemented with 2% B27 supplement (GIBCO, ThermoFisher Scientific, Grand Island, NY, USA), 20 ng/mL recombinant human basic fibroblast growth factor (GIBCO, ThermoFisher Scientific, Grand Island, NY, USA), 20 ng/mL recombinant human epidermal growth factor (GIBCO, ThermoFisher Scientific, Grand Island, NY, USA), 2% insulin‐transferrin‐selenium (GIBCO, ThermoFisher Scientific, Grand Island, NY, USA), 0.5 g/mL hydrocortisone (Sigma‐Aldrich, Merck, Darmstadt, Germany), and 1% methylcellulose (Sigma‐Aldrich, Merck, Darmstadt, Germany). After incubation, the culture was washed as described above to remove leukemic cells. MSC were collected, centrifuged at 500× *g* for 5 min and seeded at 1.5 × 10^4^ cells/well in sphere‐inducing medium and ultralow‐adherent plates (Stem Cell Technologies, Cambridge, MA, USA). After 5 days of incubation at 37ºC and 5% CO_2_ with reduced manipulation in order to prevent aggregation, mesenspheres were measured, counted (counting only those with a >70 μm diameter), and photographed with an inverted microscope (Eclipse Model TS‐100, Nikon) and an Axiovert 40 CFL Zeiss fluorescence microscope.

### 4.9 Multipotent differentiation capacity evaluation of MSC from LN

The capacity of MSC to differentiate to osteoblasts, adipocytes and chondrocytes after treatment with LN CM or co‐culture with leukemic cells was assessed as described above. First, MSC were cultured alone, with REH LN CM, SUP‐B15 LN CM, REH or SUP‐B15 cells as described above for 72 h at 37ºC and 5% CO_2_. The supernatant was discarded, and leukemia cells were removed as previously described, then, induction medium for each lineage was added to the cultures and cultured as indicated. After staining, for both osteoblasts and adipocytes, macro‐ and microscopic photographs were taken and analyzed using Image J software (National Institute of Health, USA) for quantification of stained area of each well. Staining of chondrocytes was performed with 0.1% Safranin O (Sigma‐Aldrich, St. Louis, MO, USA) at RT for 15 min; then, the staining solution was removed, and cultures were washed with PBS 1×. Photographs were taken using an inverted microscope followed by dissolving the stain using dimethyl sulfoxide (DMSO). Subsequently, the absorbance at 550 nm was measured in a spectrophotometer (Ultramark, Bio‐Rad, CA, USA).

### 4.10 Evaluation of senescence‐associated β‐galactosidase (SA‐β‐GAL) activity in MSC

Cellular senescence was assessed using the Cellular Senescence Assay Kit (KAA002, Millipore, Merck, Darmstadt, Germany) for colorimetric detection of MSC positive for SA‐β‐GAL. MSC were cultured with REH or SUP‐B15 cells, REH LN CM, SUP‐B15 LN CM, or CCL2 (Biolegend, San Diego, CA, USA) at different concentrations (12.5, 25 and 50 ng/mL) at 37ºC and 5% CO_2_ for 3–9 days, depending on the experiment. Then, the supernatant was discarded, and leukemic cells were removed as previously described; the culture was fixed for 10 min with 10% formaldehyde at RT and washed once with PBS 1×. Next, the SA‐β‐GAL detection solution containing the substrate (X‐gal) was prepared in acidic conditions (pH = 6) following the manufacturer's instructions. The microplate was sealed with parafilm and incubated in the dark at 37ºC for 4 h. The detection solution was removed, and the culture was washed once with PBS 1×. 10 randomly images per well were taken using an inverted microscope and the ImageJ software was used to perform the quantification of SA‐β‐GAL positive cells.

### 4.11 SASP characterization of LN CM

Pro‐inflammatory cytokine secretion of MSC, SUP‐B15 cells, SUP‐B15 LN and REH LN was evaluated using the Proteome Profiler Human Cytokine Array Kit (ARY005 B, R&D Systems, Minneapolis, MN, USA) according to the manufacturer's instructions. Briefly, 6 × 10^4^ MSC were seeded in a 24‐well microplate with supplemented IMDM medium and incubated at 37ºC and 5% CO_2_ for 24 h. Cultures were washed once with incomplete IMDM and 3 × 10^5^ REH cells or 4.2 × 10^5^ SUP‐B15 cells were seeded on top of the MSC monolayer in IMDM with 1% FBS. After 24 h of incubation at 37ºC and 5% CO_2_ supernatants were collected, centrifuged at 500× *g* for 5 min, and filtered using a 0.2 μm pore syringe filter. Then, the arrays were incubated with 500 μL of supernatant and the antibody cocktail provided in the kit overnight at 4ºC. Membranes were washed as indicated by the manufacturer and exposed to film between 4 and 10 min, until the spots were clearly appreciable when revealed in the medical film processor (SRX‐101A, Konica Minolta). The photograph acquisition was performed using Gene Tool software (Syngene, Frederick, MD, USA) in a Gel Imagen System (GeneGenius, Syngene, CA, USA) and ImageJ software was used for the dots analysis.

### 4.12 Cytotoxicity evaluation of MSC and leukemic cells alone or in the LN

The cell viability of MSC, REH and SUP‐B15 cells alone or in the LN after different drug treatments was assessed spectrophotometrically using the MTT assay (ThermoFisher Scientific, Portland, OR, USA). For REH and SUP‐B15 cell lines, 2.5 × 10^4^ cells seeded 96‐well microplates and treated with DOX (37 and 88 nM), MTX (22 and 56 nM), VCR (7 and 20 nM), DEX (153 and 306 μM), ASP (20 and 40 μM) (all drugs from Sigma‐Aldrich, St. Louis, MO, USA) or PDN (20–40 μM) (Santa Cruz Biothecnology, Dallas, TX, USA) for 48 h. As controls, cells were treated with IMDM +1% FBS or the vehicle at the concentration used with the respective drug. Next, MTT reagent (5 mg/mL) was added to each well and incubated during 4 h at 37 ºC and 5% CO_2_. Then, microplates were centrifuged at 1600× *g* for 10 min, supernatant was discarded and 100 μL of DMSO were added to dissolve the Formazan crystals. Subsequently, absorbance at 570 nm was measured using a spectrophotometer (Ultramark, Bio‐Rad, Hercules, CA, USA) and cell viability was calculated as a percentage of control cells.

For LN, 5 × 10^3^ MSC, cells were seeded in 96‐well microplates and pre‐treated or not (depending on the experiment) for 72 h with CCL2 (50 ng/mL), REH LN CM, SUP‐B15 LN CM or treated with MSC CM or IMDM + 1% FBS as controls. Then, supernatants were discarded and 2.5 × 10^4^ REH or SUP‐B15 cells were seeded followed by drug treatments (DOX, MTX or VCR) and incubated for 48 h at 37ºC and 5% CO_2_. For our co‐culture experiments we kept LN cultures incubating at 37ºC for 16 h after MTT reagent was added, as we have done before.^14^ For MSC alone, cells were treated with the maximum concentrations of DOX (88 nM), MTX (56 nM) or VCR (20 nM), and MTT incubation was maintained for 16 h.

### 4.13 Verification of the protective effect of LN on leukemic cells

To confirm the protective effect of the LN on REH and SUP‐B15 cells and rule out drug absorption by the stromal cell support, we pre‐incubated MSC or adipocytes with the drugs used in LN (DOX, MTX or VCR) and then used the incubated drugs to treat REH and SUP‐B15 cells. First, we established MSC and adipocytes as previously described, then, cells were treated with DOX, MTX or VCR for 48 h at 37ºC and 5% CO_2_. In order to rule out drug degradation as well, each of the three drugs were incubated with no cells in the same conditions. Next, the supernatants were collected and added to 2.5 × 10^4^ REH or SUP‐B15 cells and incubated for another 48 h at 37ºC and 5% CO_2_. As positive control, freshly prepared drugs were used. Finally, the cell viability was evaluated using the MTT assay as described above.

### 4.14 Evaluation of morphological changes of MSC from LN

For morphologic alterations, MSC were cultured with REH or SUP‐B15 cells, or treated with REH LN CM or SUP‐B15 LN CM for 48 h. The medium was discarded, and the leukemic cells were removed as described above. Then, cultures were fixed with 10% formaldehyde and stained with Wright's dye. MSC morphological changes were observed with an inverted microscope with an objective at 40×. 10 randomly taken photographs were analyzed for morphometric parameters for each individual cell using the area tool of ImageJ software.

### 4.15 Statistics

GraphPad Prism version 9.5 (GraphPad, San Diego, CA, USA) was used for statistical analyses and graphs. Data in graphs were expressed as the mean ± standard error of the mean (SEM). For the cytotoxicity, mesensphere formation, and adhesion assays, data were analyzed using a non‐parametric 1‐way analysis of variance (ANOVA) with a Kruskal–Wallis test. Comparisons between grouped conditions in the cytokine microarrays and migration assays were done using a 2‐way ANOVA test followed by Dunnett's post hoc test. The statistical significance was generated using GraphPad Prism 9.5 version software, where results were considered significant when *p* < 0.05. This significance was represented as follows, *p* = 0.05 (*), *p* = 0.01 (**), *p* = 0.001 (***), *p* = 0.0001 (****), and not significant (*ns*).

## AUTHOR CONTRIBUTIONS

**Santiago Ángel Cortés**: Methodology; formal analysis; investigation; writing—original draft preparation. **Paula‐Manuela Rojas Zambrano**: Methodology; formal analysis; investigation; writing—review and editing. **Jean‐Paul Vernot**: Conceptualization; methodology; formal analysis; investigation; funding acquisition; resources; supervision; writing—original draft; writing—review and editing.

## CONFLICT OF INTEREST STATEMENT

The authors declare no conflicts of interest.

## ETHICS STATEMENT

The study was conducted according to the guidelines of the Declaration of Helsinki and approved by the Ethics Committee of the Facultad de Medicina, Universidad Nacional de Colombia, (code 0073‐23 27.04. 2023).

## CONSENT TO PARTICIPATE

Informed consent was obtained from all individual participants included in the study.

## Supporting information

Supporting Information S1

Supporting Information S2

Figure S1

Figure S2

Figure S3

Figure S4

Figure S5

Figure S6

Figure S7

Figure S8

Table S1

## Data Availability

The data used to support the findings of this study are available from the corresponding author upon request.
