# Correction to Favorable and poor prognosis B‐cell precursor acute lymphoblastic leukemia subtypes reveal distinct leukemic cell properties when interacting with mesenchymal stem cells, differentially modifying their cell stemness and leukemia chemoresistance

**DOI:** 10.1002/ccs3.70068

**Published:** 2026-02-23

Ángel Cortés, S., Rojas Zambrano, P.‐M. and Vernot, J.‐P. (2025), Favorable and poor prognosis B‐cell precursor acute lymphoblastic leukemia subtypes reveal distinct leukemic cell properties when interacting with mesenchymal stem cells, differentially modifying their cell stemness and leukemia chemoresistance. J. Cell Commun. Signal, 19: e70009. https://doi.org/10.1002/ccs3.70009.

In the originally published article, the authors' names were included incorrectly. The correct names are mentioned below. The online version of this article has been corrected.

We apologize for this error.

Incorrect:

Ángel Cortés Santiago, Rojas Zambrano Paula‐Manuela, Vernot Jean‐Paul

Correct:

Santiago Ángel Cortés, Paula‐Manuela Rojas Zambrano, Jean‐Paul Vernot

