# Supplementary material for: Favorable and poor prognosis B‐cell precursor acute lymphoblastic leukemia subtypes reveal distinct leukemic cell properties when interacting with mesenchymal stem cells, differentially modifying their cell stemness and leukemia chemoresistance
Source: J Cell Commun Signal. 2025 Jun 12;19(2):e70009. doi: 10.1002/ccs3.70009 (PMC12162153; doi:10.1002/ccs3.70009)
Supplement: Supplementary file 1 — Supporting Information S1 [file CCS3-19-e70009-s006.docx]

**Supplementary table 1.** Drugs and their IC_50_ and double IC_50_ against SUP-B15 and REH cell lines used in experiments.

**Supplementary fig 1.** Phenotypic and functional characterization of MSC isolated from umbilical cord blood. (**a**) Flow cytometry was used to determine the cell surface expression of CD90, CD44, CD73 and CD105 in MSC and the lack of expression of CD34 and CD45 (blue histograms). Unlabelled MSC were used as controls (red histograms). (**b**) Multipotent differentiation capacity of MSC into osteogenic, adipogenic and chondrogenic lineages was assessed by culturing them in specific media for inducing differentiation during 14-21 days. Then, cultures were stained using NBT/BCIP, for osteogenic lineage; Safranin O, for chondrogenic lineage; and Oil Red, for adipogenic lineage; cells were microphotographed at 10X magnification (scale bar, 100 µm)

**Supplementary fig. 2** MSC induce an increase in cell proliferation of SUP-B15 and REH cell lines during the first 48 h. Cell proliferation during 48 and 72 h was assessed by flow cytometry using CFSE staining of SUP-B15 and REH cell lines. (**a**) Comparison between SUP-B15 cells from LN and monocultures. (**b**) Comparison between REH cells from LN and monocultures. Non-stained cells were used as negative stain control. Freshly stained cells (0 h) were used as positive stain control. Left panels show representative experiments. Right panels show the comparison of MFI of each condition. Data are expressed as the mean ± SEM of triplicates per condition. Statistical analysis was performed using one-way ANOVA test, followed by a Kruskal–Wallis test. No significant statistical differences (ns) *p*-values * < 0.05, *** < 0.001, **** < 0.0001

**Supplementary fig. 3** SUP-B15 and REH cell lines do not induce changes in MSC morphology. (**a**) MSC were co-cultured with SUP-B15 (SUP-B15 LN) or REH (REH LN) cell lines, or treated with SUP-B15 LN CM or REH LN CM for 48 h; then, cells were fixed and stained with Wright’s dye and photographed using an inverted microscope. MSC cultured alone in IMDM with 10% FBS were used as control (MSC C). Cytoplasm (C) and nucleus (N) areas (μm^2^) were determined using ImageJ software. (**b**) Microphotographs of representative experiments at 40X magnification (scale bar, 100 μm) are shown. (**c**) Bars represent the cytoplasm to nucleus (C/N) ratio. Each point on the graph represents a measurement. Statistical analysis was performed using two-way ANOVA followed by Dunnett’s post hoc test (**a**) and one-way ANOVA test followed by a Kruskal–Wallis test (**c**). No significant statistical differences (ns)

**Supplementary fig. 4** Drug susceptibility of SUP-B15 and REH cell lines. The MTT assay was used to assess leukemic cell viability. (**a**) SUP-B15 and REH cells were treated with different concentrations (10, 20, 40 and 80 nM) of DOX to determine the IC_50_ for both cell lines. Linear regressions of normalized absorbance were used to determine the IC_50_. SUP-B15 and REH cells were treated with reported IC_50_ and the corresponding double concentration of (**b**) Prednisolone (PDN), (**c**) Asparaginase (ASP) and (**d**) Dexamethasone (DEX). As controls, non-treated (NT) cells and vehicle-treated (DOX, DMSO at 0.001%; PDN, ddH_2_O at 0.015%; ASP, ddH_2_O at 0.06%; DEX, DMSO at 0.3%) cells were used. Dot lines indicate 50 % viability. Data are expressed as the mean ± SEM of triplicates per condition. Statistical analysis was performed using one-way ANOVA test, followed by a Kruskal–Wallis test. No significant statistical differences (ns) *p*-values, ** < 0.01, *** < 0.001, **** < 0.0001

**Supplementary fig. 5** MSC viability is not altered by drug treatments and MSC CM or LN CM do not protect SUP-B15 and REH cell lines against drug treatments. MTT assay was used to assess cell viability. (**a**) MSC were pre-treated with CCL2 (MSC + CCL2; 50 ng/mL) or IMDM with 1% FBS (NT MSC) for 48 h followed by treatment with MTX (56 nM), VCR (20 nM) or DOX (88 nM), corresponding to the maximum concentration of each drug used for the LN experiments. As controls, not treated cells (NT) and vehicle at the maximum concentration used (DMSO at 0.03%). (**b**) 2.5 x 10^3^ MSC seeded in a microplate followed by treatment with CCL2 or IMDM with 1% FBS for 48 h; then, cells were detached and counted using a Neubauer chamber. (**c**) SUP-B15 cell line was treated with MTX (44 nM), VCR (20 nM) or DOX (76 nM), drugs were prepared in IMDM, MSC CM or SUP-B15 LN CM. As controls, the maximum vehicle concentration (DMSO at 0.03%) was prepared in MSC CM or SUP-B15 LN CM. (**d**) REH cell line was treated with MTX (56 nM), VCR (14 nM) or DOX (88 nM), drugs were prepared in IMDM, MSC CM or REH LN CM. As controls, the maximum vehicle concentration (DMSO at 0.03%) was prepared in MSC CM or REH LN CM. Data are expressed as the mean ± SEM of triplicates per condition. Statistical analysis was performed using one-way ANOVA test, followed by a Kruskal–Wallis test. No significant statistical differences (ns) *p*-values, * < 0.05, ** < 0.01, *** < 0.001, **** < 0.0001

**Supplementary fig. 6** MTX, VCR and DOX cytotoxic effect is not decreased by stromal absorption. MTT assay was used to assess cell viability. (**a**, **b**) MSC or (**c**, **d**) adipogenic induced MSC were treated with MTX, VCR, DOX or vehicle as control (DMSO at 0.03%) for 48 h at 37ºC. We also incubated the drugs without cells at 37ºC for 48 h to assess the loss of cytotoxicity due solely to incubation. Then, supernatants were collected and used to treat (**a**, **c**) SUP-B15 and (**b**, **d**) REH cell lines for 48 h. Simultaneous treatments with freshly prepared drugs were carried out for comparison. Data are expressed as the mean ± SEM of triplicates per condition. Statistical analysis was performed using one-way ANOVA test, followed by a Kruskal–Wallis test. No significant statistical differences (ns) *p*-values, * < 0.05, ** < 0.01, **** < 0.0001

**Supplementary fig. 7** LN CM does not alter the multipotent differentiation capacity of MSC. MSC were treated with SUP-B15 LN CM or REH LN CM for 72 h. Then, supernatants were discarded and induction media for osteogenic, adipogenic and chondrogenic differentiation was added. Cultures were maintained for 14 days for osteogenic and adipogenic differentiation and 21 days for chondrogenic differentiation. (**a**) Staining with NBT/BCIP was used for osteogenic, (**b**) Oil Red for adipogenic and (**c**) Safranin O for chondrogenic differentiations. Stained area for osteogenic and adipogenic cultures were determined using Image J software (**a**, **b** right panels). Safranin O staining of chondrogenic cultures was solubilized using DMSO and absorbance at 550 nm was measured (**c** right panel). MSC cultured in MSC CM followed by induced differentiation were used as positive control (C+). MSC cultured in IMDM with 10% FBS instead of differentiation induction media were used as negative control (C-). Representative microphotographs (left panels) are shown at 10X magnification (scale bar, 100 µm). Data are expressed as the mean ± SEM of triplicates per condition. Statistical analysis was performed using one-way ANOVA test, followed by a Kruskal–Wallis test. No significant statistical differences (ns)

**Supplementary fig. 8** Stromal support cultures of MSC, adipogenic induced MSC and mixture (1:1) of them. MSC were induced to adipogenic differentiation for 7 or 14 days, and then non-induced MSC were added in the same proportion as the induced cells (1:1). Left panels show non-induced MSC; middle panels, adipogenic induced MSC alone; and right panels, 1:1 proportion of induced and non-induced MSC. All conditions have a total of 5 x 10^3^ cells. Microphotographs of representative experiments are shown at 10X magnifications (scale bar, 100 µm)
