# Supplementary material for: Favorable and poor prognosis B‐cell precursor acute lymphoblastic leukemia subtypes reveal distinct leukemic cell properties when interacting with mesenchymal stem cells, differentially modifying their cell stemness and leukemia chemoresistance
Source: J Cell Commun Signal. 2025 Jun 12;19(2):e70009. doi: 10.1002/ccs3.70009 (PMC12162153; doi:10.1002/ccs3.70009)
Supplement: Supplementary file 2 — Supporting Information S2 [file CCS3-19-e70009-s004.docx]

**TITLE:**

Favourable and poor prognosis B-cell precursor acute lymphoblastic leukaemia subtypes reveal distinct leukemic cell properties when interacting with mesenchymal stem cells, differentially modifying their cell stemness and leukaemia chemoresistance.

**Authors:** Ángel-Cortés Santiago^1^, Rojas-Zambrano Paula Manuela^1^, Vernot Jean-Paul^1,2,*^

**Affiliation:**

^1^ Fisiología Celular y Molecular, Facultad de Medicina, Universidad Nacional de Colombia, Bogotá 111321,

^2^ Instituto de Investigaciones Biomédicas, Facultad de Medicina, Universidad Nacional de Colombia, Bogotá 111321, Colombia

* Correspondence: jpvernoth@unal.edu.co; Telephone: +57-1-316-5466

**Supplementary table 1.** Drugs and their IC_50_ and double IC_50_ against SUP-B15 and REH cell lines used in experiments.
